# Nitric Oxide Made a Major Contribution to the Improvement of Quality in Button Mushrooms (*Agaricus bisporus*) by the Combined Treatment of Nitric Oxide with 1-MCP

**DOI:** 10.3390/foods11193147

**Published:** 2022-10-10

**Authors:** Xiaoyu Wang, Zhifeng Yang, Jinxia Cui, Shuhua Zhu

**Affiliations:** 1 Department of Horticulture, College of Agriculture, Shihezi University, Shihezi 832003, China; 2 Key Laboratory of Special Fruits and Vegetables Cultivation Physiology and Germplasm Resources Utilization of Xinjiang Production and Construction Crops, Shihezi 832003, China; 3 College of Chemistry and Material Science, Shandong Agricultural University, Taian 271018, China

**Keywords:** *Agaricus bisporus*, nitric oxide, 1-methylcyclopropene, antioxidant system

## Abstract

Browning is one of the major effects of shelf-life responsible for the reduction in the commercial value of the button mushrooms (*Agaricus bisporus*). In this study, the individual and the combined effects of exogenous sodium nitroprusside (SNP, a nitric oxide donor) and 1-methylcyclopropene (1-MCP) on the quality of button mushrooms were evaluated. The results demonstrated that mushrooms treated with SNP+1-MCP promoted reactive oxygen species (ROS) metabolism thereby protecting cell membrane integrity, hindering polyphenol oxidase (PPO) binding to phenolic compounds, and downregulating the PPO activity. In addition, the SNP+1-MCP treatment effectively maintained quality (firmness, color, total phenol, and flavonoid) and mitigated oxidative damage by reducing ROS accumulation and malondialdehyde production through the stimulation of the antioxidant enzymes activities and the enhancement of ascorbic acid (AsA) and glutathione (GSH) contents. Moreover, the correlation analysis validated the above results. The SNP+1-MCP treatment was observed to be more prominent on maintaining quality than the individual effects of SNP followed by 1-MCP, suggesting that the combination of NO and 1-MCP had synergistic effects in retarding button mushrooms senescence, and NO signaling molecules might be predominant in the synergy.

## 1. Introduction

*Agaricus bisporus* is regarded as a delicacy due to its unique flavor (umami), richness in protein, dietary fiber, vitamins, minerals, and amino acids. It is popular among consumers not only due to its high nutritional and medicinal values, but also due to its high return on sustainable economic and ecological value. However, the shelf life of *Agaricus bisporus* is limited due to its special epidermal structure (no protective cuticle) and high susceptibility to microbial infestation [1]. *Agaricus bisporus* has very high-water content (more than 90%) and high respiration rate, which makes it prone to irreversible physiological phenomena, such as water loss and wilting, tissue browning, and membrane breaking and opening after harvesting [2,3]. Chemical preservation has the advantage of being fast and efficient. The use of chemicals is of concern and involves the environment and human health. Therefore, finding a safe and environmentally friendly means of chemical preservation is essential.

Nitric oxide (NO) and 1-methylcyclopropene (1-MCP) are widely used to delay the senescence of fruit and vegetables due to their superior fresh-keeping effect and safety without residues, such as pear [4], cauliflower [5], mango [6], and persimmon [7]. As a biologically active small molecule, NO plays an important role in regulating fruit storage quality and after-ripening. NO can reduce oxidative stress by directly scavenging ROS in plants or by stimulating antioxidant defense systems, thereby maintaining the sensory and nutritional quality of fruits [8]. On the other hand, it delays the senescence of fruits and vegetables by inhibiting the biosynthesis of ethylene [9,10]. Explosions of ethylene can cause physiological disruptions, rapid senescence, and susceptibility to pathogens, which in turn shorten shelf life [11]. Zhu et al. [12] found that NO can combine with 1-aminocyclopropane-1-carboxylate oxidase (ACO) to form a binary ACO-NO complex. Then, the binary complex is chelated by 1-aminocyclopropane-1-carboxylate (ACC) to form a stable ACC-ACO-NO ternary complex, which cannot be converted into ferric species that affect the oxidation of ACC to ethylene, further inhibiting ethylene synthesis. 1-MCP is an ethylene inhibitor that strongly binds to the ethylene receptor through a double bond, preventing the normal binding of ethylene to its receptor and resulting in blocked transmission and expression of ethylene action signals [13]. NO requires metal cofactors to transport the hormone ethylene to bind to fruit and vegetable receptors, while 1-MCP acts on ethylene receptors, thus it is speculated that the relationship between NO and 1-MCP could enhance their ability to delay vegetable senescence [14].

The combination of exogenous NO with 1-MCP to preserve the quality of vegetables has been reported in blueberries and tomatoes [15,16], but has not been studied in the preservation of *Agaricus bisporus*. The single preservation technology method has not achieved a satisfactory effect. The synergistic use of preservation technologies will be the main trend in post-harvest preservation of fruit and vegetables. For example, the combination of 1-MCP and 5% CO_2_ in cold storage can store persimmons for up to 4 months while maintaining high nutritional and antioxidant capacity compared with 1-MCP alone [7]. Gholami et al. [17] found that the combination of nano-doped packaging film and chitosan coating treatment extended the storage period of *Agaricus bisporus* at 4 °C for up to 22 d. Therefore, the purpose of this work was to evaluate the effect of *Agaricus bisporus* postharvest treatment with 1-MCP and NO individually or in combination, in order to provide practical basis and theoretical reference for the application of fresh-keeping of *Agaricus bisporus*.

## 2. Materials and Methods

### 2.1. Plant Materials and Treatment

Mushrooms (*Agaricus bisporus*, W192) were freshly picked from Taian, China. Mushrooms of the approximate size and excellent quality were selected and randomly divided into three piles as three replicates. Each pile (about 21 kg per pile) was then randomly divided into two groups and treated with 0 and 5 mg L^−1^ of 1-MCP in six separated 400 mm desiccators for 16 h at 25 °C. Then, each group was divided into two treatments: 6 L of distilled water or 10 μmol L^−1^ of SNP solution. The groups were immersed in the solution for 5 min, respectively. After air drying, the mushrooms were stored at 25 ± 1 °C and 75–90% relative humidity (RH) for 6 d. 1-MCP (CAS No. 3100-04-7) and SNP (CAS No. 13755-38-9) were purchased from Shanghai Yuanye and Macklin Bio-Technology Co. Ltd., Shanghai, China, respectively. At least 40 mushrooms were randomly selected at 9:00 a.m. daily. The color and firmness were measured with at least 0.5 kg mushrooms from each treatment. The rest of the mushrooms were frozen in liquid nitrogen and stored at −80 °C.

### 2.2. Firmness and Color Measurements

The firmness of the mushroom cap was measured by a penetration test using GY-4 fruit hardness tester (Shanghai Shandu Co., Shanghai, China) equipped with an 11 mm diameter probe at room temperature, and expressed in Newton.

The surface color of the mushroom caps was measured using a color reader (CR-10, made in Japan) and used ΔE as described by the equation as follows [18]:

ΔE = ((L^*^ − *L*_0_^*^)^2^ + (a^*^ − *a*_0_^*^)^2^ + (b^*^ − *b*_0_^*^)^2^)^1/2^

where “L^*^” shows light and dark, “a^*^” shows red and green, and “b^*^” shows yellow and blue. The subscript “0” indicates the data measured from the sample on day 0.

### 2.3. Electrolyte Leakage (EL) Measurement

The electrolyte leakage rate is an important parameter reflecting the degree of cell membrane damage. When plants are under adverse conditions, adverse environmental factors first act on the plasma membrane, causing damage to the plasma membrane and an increase in membrane permeability. Stressed plant tissues were immersed in deionized water, electrolytes were extravasated, and then the conductivity of water increased. Therefore, the conductivity meter could be used to determine the change in plasma membrane permeability by measuring the change in conductivity of the extracellular fluid [19]. The specific steps are listed below. Ten mushrooms were randomly taken from each treatment, cut into three 2-mm slices, and were floated in 40 mL of deionized water. Then, EL was timely recorded (P_0_). The EL was measured once after incubation at room temperature for 10 min and recorded as P_1_. Subsequently, the sample was boiled for 10 min. After cooling to 25 °C, the last EL (P_2_) was documented. EL could be calculated according to the following formula:

EL= ((P_1_ − P_0_)/(P_2_ − P_0_)) × 100%

### 2.4. Malondialdehyde (MDA) Content Measurement

MDA content was determined by referring to Zhang et al. [20]. Mushroom powders (2 g) were homogenized with 6 mL of 10% (*w*/*v*) tri-chloroacetic acid (TCA), and the homogenate was centrifuged at 13,000× *g* and 4 °C for 20 min. The supernatant (1 mL) was added to 2 mL of 15% (*w*/*v*) TCA containing 0.5% (*w*/*v*) thiobarbituric acid. After heating in a boiling water bath for 30 min, the mixture was cooled in time and centrifuged at 12,000× *g* at 4 °C for 10 min. Absorbencies were measured at 450, 532, and 600 nm. The MDA content was calculated according to the following equation and expressed as mmol g^−1^ on the basis of fresh weight:

MDA (μmol·g^−1^) = 6.45 × (A_532_ − A_600_) − 0.56 × A_450_

### 2.5. Superoxide (O_2_^·−^), Hydrogen Peroxide (H_2_O_2_), and Hydroxyl Radical (·OH) Content Measurements

The O_2_^·−^ content was measured following the method by Zhang et al. [21]. Briefly, 1 mL of extract was incubated with 4 mL of reaction mixture containing hydroxylamine hydrochloride (10 mmol L^−1^), sulfanilic acid (17 mmol L^−1^), and α-naphthol (7 mmol L^−1^) at 30 °C for 1 h, and the absorption was recorded at 525 nm using a UV-6100S spectro-photometer. The result was expressed as nmol g^−1^ on the basis of fresh weight.

The content of H_2_O_2_ was determined according to the method described by Zhu et al. [22]. Mushroom powders (2 g) were mixed with 6 mL of precooled acetone. The homogenate was centrifuged at 12,000× *g* for 15 min at 4 °C. The extracted solution (1 mL) was mixed with 0.1 mL of 5% Ti(SO_4_)_2_ and 0.2 mL of concentrated NH_4_OH solution. The mixture was centrifuged at 4 °C for 10 min at 12,000× *g*, and the supernatant was discarded, then acetone was added 3–5 times to reduce color interference. The absorbance value was measured at 415 nm by dissolving the precipitate in 3 mL of 18 mol L^−1^ H_2_SO_4_. The H_2_O_2_ content was calculated by comparison with a standard with known H_2_O_2_ concentrations and expressed as nmol g^−1^ on the basis of fresh weight.

The production of ·OH was measured by referring to Shi et al. [10]. The sample (1 g) was homogenized with 1.2 mL of 50 mmol L^−1^ sodium phosphate buffer (pH 7.0) and centrifuged at 10,000× *g* for 20 min at 4 °C, and then 0.5 mL of supernatant was mixed with 0.5 mL of 50 mmol L^−1^ sodium phosphate buffer (pH 7.0) and 1 mL of 25 mmol L^−1^ sodium phosphate buffer (pH 7.0) containing 2.5 mmol L^−1^ 2-deoxyribose. The reaction was developed at 35 °C in the dark for 1 h. Then, 1 mL of 1% TBA in 0.05 mmol L^−1^ NaOH and 1 mL of acetic acid were added. The mixture was boiled for 30 min and immediately cooled for 10 min on ice. The production of ·OH was followed by the measurement of absorbance at 532 nm, and the content of ·OH was expressed as absorbance g^−1^ on the basis of fresh weight.

### 2.6. Enzyme Extraction and Assays

Superoxide dismutase (SOD) and catalase (CAT) activities were determined as described in the method by Wang et al. [23]. Peroxidase (POD) and polyphenol oxidase (PPO) activities were determined as described in the method by Hu et al. [24]. One unit (U) of SOD, CAT, and POD activities were defined as the amount of enzyme converting 1 μmol of substrate per second under assay conditions, and expressed as U mg^−1^ protein. One unit of PPO activity was defined as the amount of enzyme required to decrease the absorbance by 0.001 per second, and the result was expressed as U mg^−1^ protein.

Ascorbate peroxidase (APX), glutathione reductase (GR), monodehydroascorbate reductase (MDHAR), and dehydroascorbate reductase (DHAR) activities were determined as described in the method by Hasanuzzaman et al. [25]. Glutathione peroxidase (GPX) activity and the protein content were determined according to the method by Huan et al. [26]. One unit (U) of APX, GR, MDHAR, DHAR, and GPX activities were defined as the amount of enzyme converting 1 μmol of substrate per second under assay conditions, and expressed as U mg^−1^ protein.

The protein content of the extracts of the above enzymes was determined using the method by Bradford [27].

### 2.7. Total Phenolic and Total Flavonoid Contents Measurements

Approximately 2.0 g of frozen samples were ground in liquid nitrogen and homogenized with 6 mL of 60% precooled ethanol. After centrifugation at 12,000× *g* for 15 min, the supernatant was collected for the following assay.

Total phenolic and flavonoid contents were measured according to the method by Nie et al. [28]. The supernatant (0.1 mL) was mixed with 0.3 mL of 0.5 mol L^−1^ Folin-Ciocalteu reagent, followed by 1.2 mL of 0.5 mol L^−1^ Na_2_CO_3_ solution, and then incubated at room temperature for 1 h in the dark. Thereafter, the absorbance was measured at 760 nm with a UV-spectrophotometer (UV-6100S, Shanghai General Instrument Co., Ltd., China). Gallic acid was used to calculate the standard curve, and total phenolic content was expressed as gallic acid equivalents (mg g^−1^).

The supernatant (0.2 mL) was mixed with 2 mL of distilled water, followed by the addition of 0.04 mL of 5% (*w*/*v*) Na_2_CO_3_ solution and 0.16 mL of 10% (*w*/*v*) Al(NO_3_)_3_ solution, then incubated at room temperature for 15 min. The absorption was recorded at 510 nm. Flavonoid content was calculated as mg of rutin equivalents per g.

### 2.8. The Ascorbic Acid (AsA) and Glutathione (GSH) Contents Measurements

AsA and GSH contents were determined as described in the method by Huang et al. [29]. Results were expressed as mg g^−1^ on the basis of fresh weight.

### 2.9. Statistical Analysis

All experimental results were derived from three replications. All the data passed the Shapiro-Wilk test and Levene’s test to check the normal distribution and the homogeneity of variance. Then, data were subjected to the analysis of variance (ANOVA). Comparison of differences between pairs of means was conducted by Duncan’s test. The variation with statistical significance was considered if *p* < 0.05.

## 3. Results

### 3.1. Firmness, Lightness(L^*^), EL, and MDA Content

**Figure 1:**
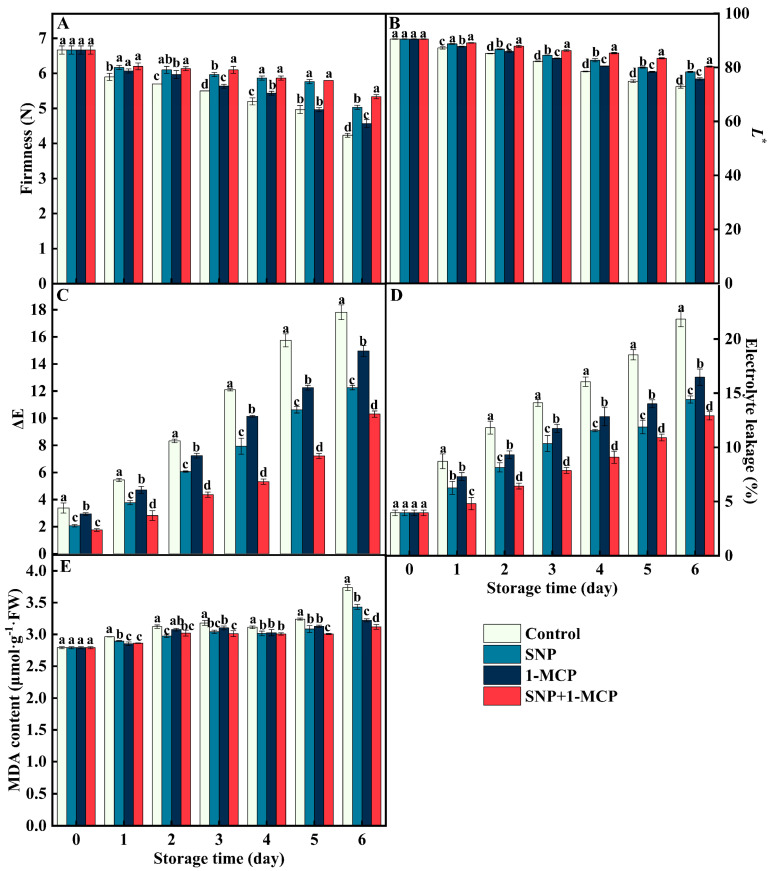
(**A**) Firmness, (**B**) L^*^, (**C**) ΔE, (**D**) electrolyte leakage (EL), and (**E**) malondialdehyde (MDA) content of *Agaricus bisporus* at 25 °C. The letters indicate the significant difference (*p* < 0.05), and the bars indicate standard error (means ± SE).

The firmness and L^*^ of mushrooms showed a decreasing trend, while the ΔE, EL, and MDA content showed an increasing trend in mushrooms during storage (Figure 1). Specifically, the SNP+1-MCP treatment significantly (*p* < 0.05) sustained high firmness and white color (Figure 1A,B) and suppressed mushroom ΔE, EL, and MDA levels (Figure 1C,D). The SNP, 1-MCP, and SNP+1-MCP treatments maintained firmness (118.91%, 107.80%, and 126.01%), L^*^ (107.54%, 103.89%, and 110.20%), ΔE (68.77%, 83.94%, and 57.83%), EL (66.07%, 75.49%, and 59.17%), and MDA content (91.85%, 86.22%, and 83.48%) in mushrooms compared with the control on day 6. Furthermore, 1-MCP-treated maintenance on the quality of mushrooms were lower than observed with SNP or SNP+1-MCP treatment (Figure 1).

### 3.2. Contents of O_2_^·−^, H_2_O_2_, and ·OH

**Figure 2:**
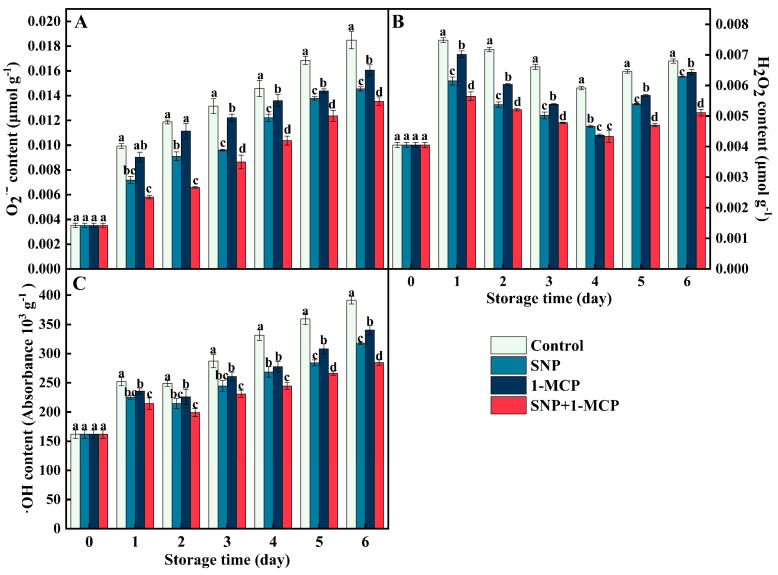
(**A**) Superoxide (O_2_^·−^), (**B**) hydrogen peroxide (H_2_O_2_), and (**C**) hydroxyl radical (·OH) contents of *Agaricus bisporus* at 25 °C. The letters indicate the significant difference (*p* < 0.05), and the bars indicate standard error (means ± SE).

The O_2_^·−^ content of *Agaricus bisporus* displayed an overall increasing trend during storage, and the other three groups significantly (*p* < 0.05) constrained the increase in O_2_^·−^ content compared with the control (Figure 2A). The SNP+1-MCP treatment significantly (*p* < 0.05) exhibited a better inhibitory effect than SNP or 1-MCP individually on days 5 and 6. In addition, the SNP+1-MCP treatment maintained a low level of H_2_O_2_ in *Agaricus bisporus*, which was only 75.19% of the control (Figure 2B). On day 1, the highest level of H_2_O_2_ content was found in mushrooms, but the H_2_O_2_ content reached the lowest level on day 4. Similar inhibitory effects of the SNP+1-MCP treatment were also found in the content of ·OH (Figure 2C). There was an interesting result that the treatment with SNP had inhibition successfully on the contents of O_2_^·−^, H_2_O_2_, and ·OH than the mushrooms treated with 1-MCP, although the inhibitory effects of SNP treatment were lower than the SNP+1-MCP treatment, suggesting that the SNP treatment had a higher ability in reducing ROS injury than the 1-MCP treatment.

### 3.3. Activities of SOD, CAT, POD, and PPO

**Figure 3:**
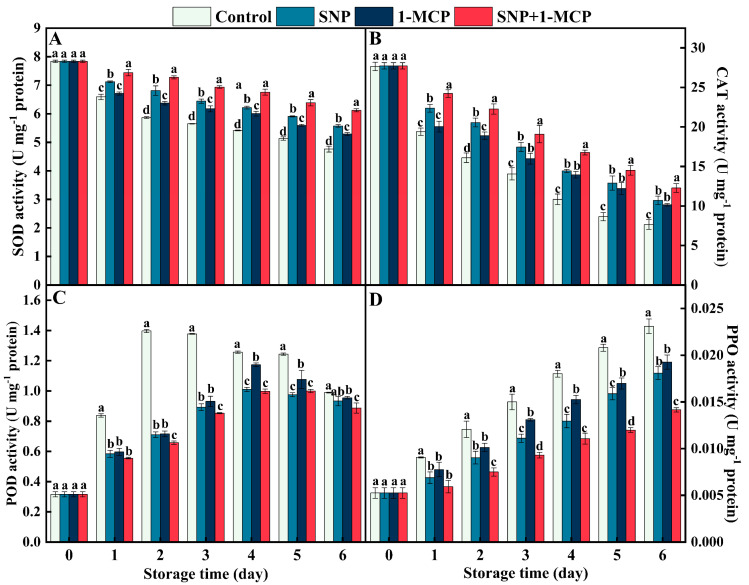
(**A**) Superoxide dismutase (SOD), (**B**) catalase (CAT), (**C**) peroxidase (POD), and (**D**) polyphenol oxidase (PPO) activities of *Agaricus bisporus* at 25 °C. The letters indicate the significant difference (*p* < 0.05), and the bars indicate standard error (means ± SE).

An overall decreasing tendency was shown in the activities of SOD and CAT in mushrooms, while the activity of PPO was opposite (Figure 3A,B). Compared with the control, SNP+1-MCP treatment significantly (*p* < 0.05) increased the activities of SOD and CAT and inhibited the activity of PPO. In addition, SNP+1-MCP treatment effect is better than the SNP treatment, whereas the SNP treatment is better than the 1-MCP treatment. POD activity increased sharply in the 1–2 d with a slow decrease after day 3 (Figure 3C). Compared with the control, the other treatments significantly (*p* < 0.05) inhibited POD activity. Particularly on the second day of storage, POD activity of SNP, 1-MCP, and SNP+1-MCP-treated mushrooms was 50.83%, 51.26%, and 47.08% of the control, respectively.

### 3.4. The Ascorbate–Glutathione Cycle (AsA-GSH)-Related Enzyme Activity

**Figure 4:**
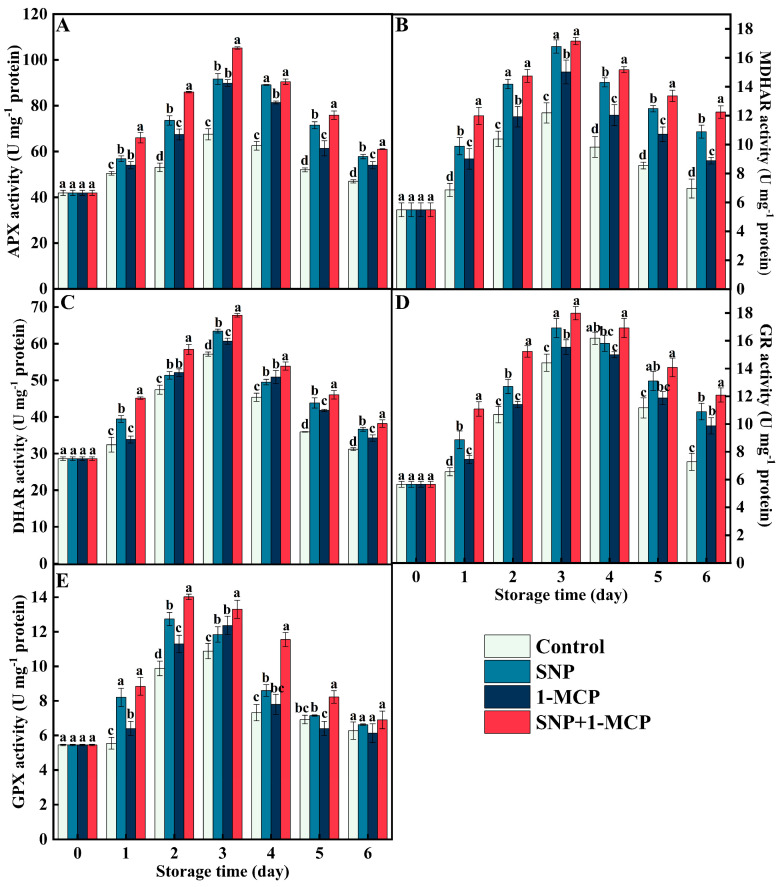
(**A**) Ascorbate peroxidase (APX), (**B**) monodehydroascorbate reductase (MDHAR), (**C**) dehydroascorbate reductase (DHAR), (**D**) glutathione reductase (GR), and (**E**) glutathione peroxidase (GPX) activities of *Agaricus bisporus* at 25 °C. The letters indicate the significant difference (*p* < 0.05), and the bars indicate standard error (means ± SE).

In the storage process, the activities of APX, GR, MDHAR, DHAR, and GPX in mushrooms showed an increasing trend followed by a decreasing trend (Figure 4). The highest peaks of APX, MDHAR, and DHAR activities all occurred on day 3. Interestingly, compared with the control, the peak GR and GPX activities appeared one day earlier in the SNP-treated and SNP+1-MCP-treated mushrooms, while the peak GPX activity appeared simultaneously in the control and 1-MCP treatment. The APX, MDHAR, and DHAR activities in mushrooms treated with SNP+1-MCP were significantly (*p* < 0.05) higher than SNP and 1-MCP treatments (Figure 4A–C). Compared with the control, SNP, 1-MCP, and SNP+1-MCP treatments showed increases in APX activity (135.71%, 133.10%, and 155.88%), MDHAR activity (137.66%, 123.24%, and 140.71%), and DHAR activity (110.98%, 106.13%, and 118.55%) on day 3 (Figure 4A–C). Moreover, GR and GPX activities showed the same trend as APX, MDHAR, and DHAR activities. In the pre-storage period, the GR and GPX activities of SNP+1-MCP-treated mushrooms were significantly higher (*p* < 0.05) than the SNP treatment (except on day 1), and the SNP treatment was significantly higher than the 1-MCP treatment. The GR activity of SNP, 1-MCP, SNP+1-MCP-treated mushrooms was 1.19, 1.06, and 1.43 times the control on day 2, respectively. Furthermore, compared with the control, SNP, 1-MCP, SNP+1-MCP treatment enhanced the GPX activity by 128.88%, 114.29%, and 141.91% on day 2, respectively.

### 3.5. Contents of Antioxidant Substances

**Figure 5:**
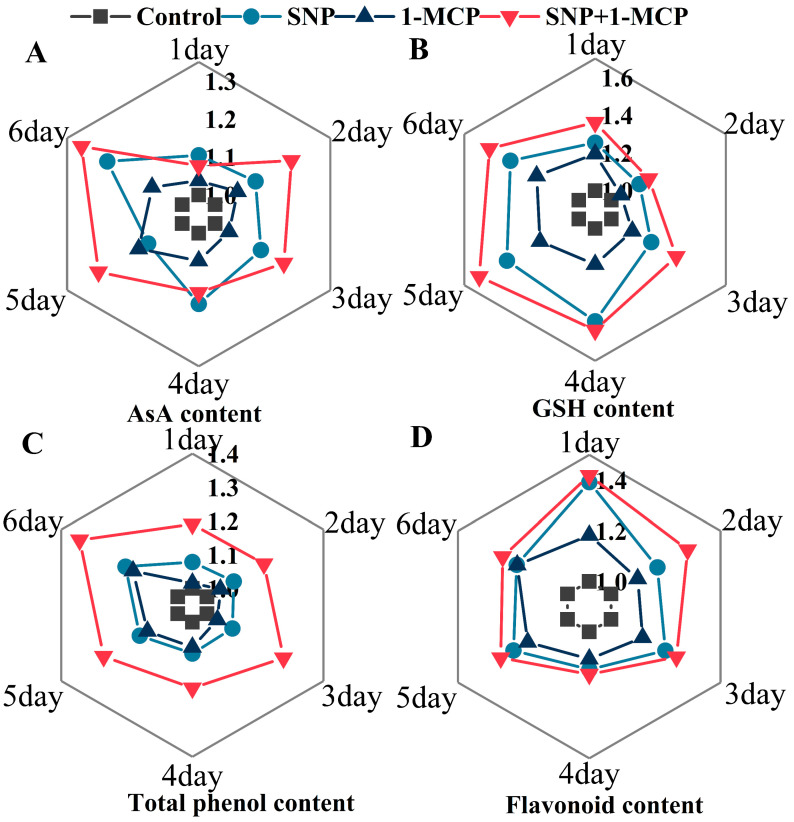
(**A**) Ascorbic acid (AsA), (**B**) glutathione (GSH), (**C**) total phenol, and (**D**) flavonoid contents of *Agaricus bisporus* at 25 °C.

AsA, GSH, total phenol, and flavonoid are the non-enzymatic antioxidants in plants. All treatments promoted the accumulation of AsA, GSH, total phenol, and flavonoid contents (Figure 5). The AsA content of mushrooms treated with SNP+1-MCP was higher than the mushrooms treated with SNP (except on days 1 and 4), which were higher than the mushrooms treated with 1-MCP (except on day 5) (Figure 5A). Compared with the control, the supplementations of the SNP, 1-MCP, and SNP+1-MCP resulted in an increase in the content of AsA on day 6 by 1.22-fold, 1.09-fold, and 1.31-fold, respectively. The GSH, total phenol, and flavonoid contents of mushrooms treated with SNP+1-MCP were also higher than the mushrooms treated with SNP, which were higher than the mushrooms treated with 1-MCP (Figure 5B–D). In comparison with the untreated mushrooms, the addition of SNP, 1-MCP or SNP+1-MCP increased the GSH content (1.41-fold, 1.26-fold, and 1.55-fold), total phenol content (1.18-fold, 1.15-fold, and 1.33-fold), and flavonoid content (1.23-fold, 1.22-fold, and 1.29-fold) in the mushrooms on day 6.

### 3.6. Correlation between Quality, ROS Damage, Antioxidant Enzymes, and Antioxidants of Agaricus bisporus

**Figure 6:**
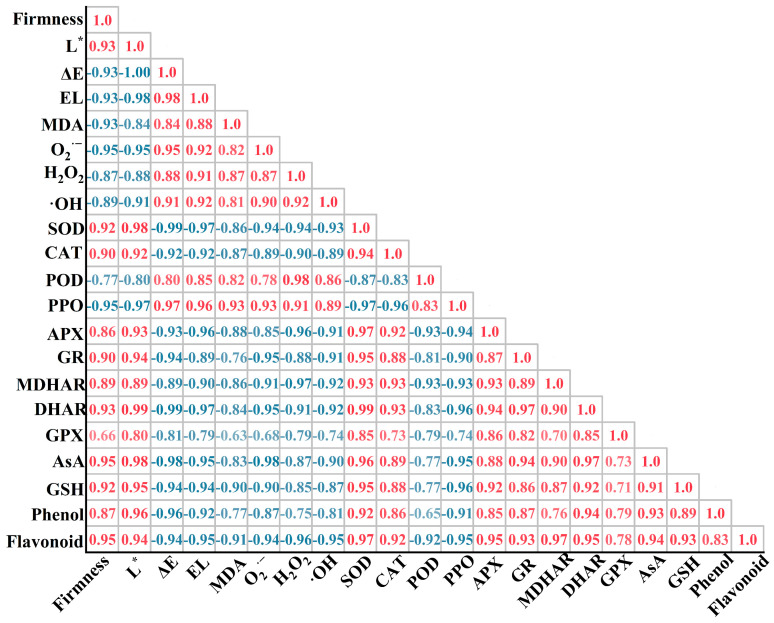
Pearson correlation coefficient matrix between each index of *Agaricus bisporus* stored at 25 °C for the sixth day. Red and blue colors represent positive and negative correlations between factors.

The results of the correlational analysis were summarized in Figure 6. Firmness was positively correlated with antioxidant enzymes (SOD, CAT, APX, GR, MDHAR, DHAR, and GPX), antioxidant substances (AsA and GSH), nutritional quality indicators (total phenols, flavonoids), and negatively correlated with ΔE, EL, MDA, and ROS (O_2_^·−^, H_2_O_2_, and ·OH). MDA was positively correlated with ROS (O_2_^·−^, H_2_O_2_, and ·OH), POD, PPO, and negatively correlated with antioxidant enzymes (SOD, CAT, APX, GR, and MDHAR), antioxidant substances (AsA and GSH), and flavonoid. Flavonoid was positively correlated with antioxidant enzymes (APX, GR, MDHAR, DHAR, and GPX), antioxidant substances (AsA and GSH), and negatively correlated with ΔE, EL, MDA content, ROS (O_2_^·−^, H_2_O_2_, and ·OH), POD and PPO activities.

## 4. Discussion

Under room temperature conditions, the apparent after-ripening effect of *Agaricus bisporus* as well as its high respiration and extremely degradable characteristics make it extremely soft and unstable for storage [1,2]. Both NO and 1-MCP can inhibit ethylene. The difference is that NO inhibits ethylene biosynthesis, while 1-MCP acts on ethylene receptors. Currently, reports on NO and 1-MCP in delaying aging in *Agaricus bisporus* are lacking. In this study, we investigated the effects of SNP and 1-MCP treatments alone and in combination on the postharvest storage performance of *Agaricus bisporus*.

During storage, the texture of *Agaricus bisporus* became rough as maturity and moisture content changed. At the same time, *Agaricus bisporus* undergoes physiological processes, such as nutrient consumption and biochemical reactions involving enzymes, and shows symptoms, such as softening and browning, thereby shortening the shelf life. Firmness and color are the main factors that people can intuitively judge the quality of *Agaricus bisporus*. The color can reflect the degree of browning of *Agaricus bisporus*. In this study, compared with CK, both SNP or 1-MCP treatments applied alone maintained the higher firmness, L^*^ and lower ΔE of *Agaricus bisporus*, and the combination of both treatments was better than the individual treatments. The results showed that the combined treatment of exogenous NO and 1-MCP had a positive effect on maintaining the storage quality of *Agaricus bisporus* (Figure 1A,B).

NO acts synergistically with 1-MCP to alleviate oxidative damage to *Agaricus bisporus*. Excessive accumulation of ROS can be triggered by handling practices, storage environments, and mechanical damage from various processes. Notably, about 1% of the O_2_ consumed by the plant is converted into a substrate for ROS production [11]. The three most common forms of ROS in plants are O_2_^·−^, H_2_O_2_, and ·OH [30]. These oxygenated substances can destroy macromolecular structures, such as proteins and nucleic acids, especially attacking unsaturated double-chain fatty acids in cell membranes. During storage, both SNP and 1-MCP reduced the levels of O_2_^·−^, H_2_O_2_, and ·OH to different degrees compared with CK, and SNP+1-MCP treatment was the most effective (Figure 2). The same changes in ROS (O_2_^·−^, H_2_O_2_, and ·OH) were found in previous studies of anxi persimmons treated with 1-MCP or table grapes treated with NO [21,31] implying that NO and 1-MCP may alleviate postharvest damage in fruits and vegetables by reducing the levels of reactive oxygen species.

NO acts synergistically with 1-MCP to maintain the integrity of the cell membrane of *Agaricus bisporus*. The phenolic is distributed in different subcellular regions with phenol oxidase (for example, PPO) under normal conditions. However, when the organism undergoes mechanical damage or natural senescence, the cell membrane integrity is disrupted, the degree of membrane lipid peroxidation is subsequently increased, regionalization will be disrupted, enzymes come into contact with the substrate, and phenols are oxidized to quinones, which results in browning [32]. Evidently, the integrity of the cell membrane plays an important role in inhibiting the development of browning. MDA content is often used as an important indicator of the degree of membrane lipid peroxidation [33]. In the present study, application of SNP or 1-MCP alone inhibited the increase in MDA, EL, and PPO activity compared with CK (Figure 1C,D and Figure 3D), and the inhibition effect was ranked as SNP+1-MCP>SNP>1-MCP>CK. This suggests that SNP+1-MCP treatment can delay browning of *Agaricus bisporus* by reducing cell membrane damage, maintaining membrane integrity, and reducing enzyme exposure to phenolics. Studies on mushrooms treated with melatonin and persimmons treated with 1-MCP also confirmed that low levels of MDA and EL were consistent with the low activity of PPO [31,34]. Moreover, in our study, the accumulation of ROS (O_2_^·−^, H_2_O_2_, and ·OH), indicators of cell membrane integrity (EL and MDA), and quality (firmness, L^*^ and ΔE) showed a close correlation and coefficients up to 0.85 or more (Figure 6), further validating the above analysis.

NO acts synergistically with 1-MCP to increase the antioxidant enzyme activity in *Agaricus bisporus*. Endogenous antioxidant systems exist in *Agaricus bisporus* to protect against oxidative stress, both enzymatically and non-enzymatically. Essential protective enzymes of the enzymatic clearance system include SOD, CAT, and POD [35]. SOD is the first line of defense of the antioxidant enzyme system, which catalyzes O_2_^·−^ disproportionation reactions to produce H_2_O and O_2_ [36]. CAT is a tetramer containing heme. CAT catalyzes the decomposition of two H_2_O_2_ molecules into water and oxygen and has a high specificity for H_2_O_2_ [35]. POD not only decomposes hydrogen peroxide into water and oxygen, but also oxidizes phenolic substances in the presence of H_2_O_2_ to induce the onset of browning. The AsA-GSH cycle is a cycle designed to remove H_2_O_2_, which consists of four enzymes (APX, GR, MDHAR, and DHAR) and two metabolites (AsA and GSH). In the AsA-GSH cycle, APX plays a key role in the scavenging of H_2_O_2_, while MDHAR, DHAR, and GR provide substrates for H_2_O_2_ scavenging by APX mainly by affecting the regeneration of AsA and GSH [30]. GPX, as a typical enzymatic ROS scavenger, can decompose hydrogen peroxide to protect organisms from oxidative damage [37]. In this study, the activities of SOD, CAT, POD, APX, GR, MDHAR, DHAR, and GPX in the fruiting bodies of *Agaricus bisporus* treated with CK were at lower levels, while the activity of PPO was the largest, implying that the ability of mushrooms to scavenge ROS decreases with the prolongation of storage time. Many studies have shown that 1-MCP and NO have different mechanisms of action, but both can delay the accumulation of oxidants and the intensification of membrane lipid peroxidation, increase the activity of antioxidant enzymes, and delay the decline of fruit and vegetable quality, for example, in tomatoes [16], blueberries [15], grapes [19], and persimmons [31]. In the present work, SNP or 1-MCP treatment upregulated SOD, CAT, POD, APX, GR, MDHAR, DHAR, and GPX activities and downregulated PPO activity compared with CK, and SNP treatment was superior to 1-MCP treatment and SNP+1-MCP treatment was superior to SNP treatment, indicating that SNP+1-MCP treatment may have a gaining effect on the activation of enzymatic antioxidant system (Figure 3 and Figure 4). Dong et al. [38] found that the 1-MCP treatment promoted the synthesis of endogenous NO in Suli pears. Therefore, we speculated that 1-MCP induced lower levels of endogenous NO than the exogenous NO supply pool (SNP). Additionally, ROS (O_2_^·−^, H_2_O_2_, and ·OH) showed a negative correlation with antioxidant enzymes (SOD, CAT, and AsA-GSH), and the correlation coefficient could reach above 0.82 (Figure 6). The correlation analysis further demonstrated that the SNP+1-MCP treatment scavenged toxic ROS by activating the antioxidant system in *Agaricus bisporus*.

NO acts synergistically with 1-MCP to maintain the antioxidant content in *Agaricus bisporus*. AsA and GSH are important indicators of the level of plant reducing reservoir power. When soybean is subjected to abiotic stress, exogenous NO positively regulates the activity of critical enzymes in the AsA-GSH cycle and the levels of AsA and GSH [39], and the same results are found in 1-MCP treatment [5]. In addition, total phenol and flavonoid, as secondary metabolites, are also good non-enzymatic antioxidants in plants [40]. The use of NO fumigation activates the biosynthetic pathways of total phenol and flavonoid and improves their antimicrobial capacity in mushrooms [41]. Li et al. [42] found that 1-MCP has the same effect on preserving pears. Studies have shown that post-harvest fruits and vegetables show a rapid increase in antioxidants followed by a rapid decrease, which is consistent with our results (CK) [11]. 1-MCP promoted the synthesis of AsA, GSH, total phenols, and flavonoids in the mushroom, and further improved the ability of scavenging reactive oxygen radicals in the mushroom (Figure 5). We speculated that this is the result of the joint action of NO and 1-MCP, but the specific molecular mechanism remains to be studied.

## 5. Conclusions

This study shows that the combined use of SNP and 1-MCP is more effective than single use in prolonging the shelf life of *Agaricus bisporus*, and SNP and 1-MCP may have a synergistic effect in maintaining the postharvest quality of *Agaricus bisporus*. SNP+1-MCP treatment of *Agaricus bisporus* can reduce the accumulation of ROS by upregulating ROS metabolism (enzymatic and non-enzymatic systems) and maintaining the integrity of cell membranes, thus impeding the binding of PPO to substrates and inhibiting the activity of PPO, effectively reducing the occurrence of browning in *Agaricus bisporus*. While alleviating the oxidative damage of *Agaricus bisporus*, SNP+1-MCP treatment effectively maintained the firmness, white color (L^*^), total phenolic, and flavonoid contents of *Agaricus bisporus*. In general, the combined application of NO and 1-MCP can be used as a potential method in the preservation of *Agaricus bisporus* at room temperature.

## Data Availability

Data are contained within the article.
